# Primary Meningococcal Type C Arthritis: A Case Report and Literature Review

**DOI:** 10.1155/2017/4696014

**Published:** 2017-04-10

**Authors:** Maximiliano Barahona, Jaime Catalan, Yoshiro Sato, Jaime Hinzpeter

**Affiliations:** Orthopaedic Department at Hospital Clínico Universidad de Chile, Santos Dumont 999, Santiago, Chile

## Abstract

Acute septic arthritis is a common clinical problem in emergency departments. Primary meningococcal arthritis (PMA) is very rare and few cases are reported in literature. D. B. M. consulted the emergency department for knee pain and fever; analysis showed that the cause was a* Neisseria meningitidis* type C infection. He received a treatment consisting of 2 arthroscopies and 5 weeks of antibiotics. At five weeks he returned to work and at 2 months he resumed sports (jogging and soccer) without complaints. Primary arthritis of the knee caused by* Neisseria meningitidis* is very rare. It has a very good response to antibiotics and arthroscopy procedure. Short-term follow-up and functional results are often good or excellent.

## 1. Introduction

Acute septic arthritis is a common clinical problem in emergency departments; the knee is the most frequently affected joint, reaching approximately 50% incidence. Despite advances in medicine, septic arthritis is still an important case of morbidity and sequelae; major problems still exist due to delay of diagnosis and lack of positive cultures [1, 2].

Classic presentation is acute loss of range of motion associated with pain and a local temperature increase. Synovial fluid analysis is the gold standard for diagnosis. The most frequent agent is* Staphylococcus aureus*; nevertheless, some presentations are unusual, those being more insidious, and therefore different agents must be expected like Gram-negative bacteria,* Mycobacterium*, and fungi. Before antibiotics,* Neisseria gonorrhoeae* was the most common cause; nowadays it must be suspected in young sexually active patients. Most cases are monomicrobial, as they spread hematogenously. Polymicrobial infections are due to penetrating injuries or contiguous spread from adjacent compartments. Independent of the efforts, about 30% of the cases have no known bacteriological diagnosis; however, this is not correlated with negative functional results [2, 3].

*Neisseria meningitidis* is a well-known bacterium for causing meningitis. Other clinical presentations are meningococcemia and primary meningococcal arthritis (PMA). Meningococcemia is associated with septic arthritis in approximately 10%; on the other hand, PMA is very rare and few cases are reported in literature [4].

We report a very rare case of primary arthritis of the knee joint in a young male caused by* Neisseria meningitidis* without evidence of meningococcemia or meningitis symptoms.

## 2. Case

 The patient, D. B. M., 19-year-old male, without known allergies, is an active consumer of cocaine and marijuana and had a single sexual partner in the last 6 months. He arrived at the emergency room, complaining about two days of knee pain, swelling, and fever. He denied a traumatic event and by his own decision he self-administered two doses of one gram of azithromycin.

Physical exams reveal an axillar temperature of 37.8 degrees, swelling +++/+++, range of motion of −20°/30° (extension/flexion), and a local temperature increase. It was decided to perform an arthrocentesis by a superolateral approach, which gives out a purulent fluid. Articular fluid analyses show 80.000 cells count and a Gram-negative diplococcus. Laboratory analysis shows White Blood Cells count of 17660, C-Reactive Protein (CRP) of 62 mg/L, and an Erythrocyte Sedimentation Rate of 17 mm/hr. No X-ray was requested.

After 3 hours of admission, arthroscopy was carried out, synovial cultures were taken, and ceftriaxone (2 grams per day) was started. 12 liters of saline solution and a shaver were used. The chondral tissue was observed to be healthy in the three knee compartments as well as the meniscus and the cruciate ligaments. No radiofrequency was used.

**Figure 1:**
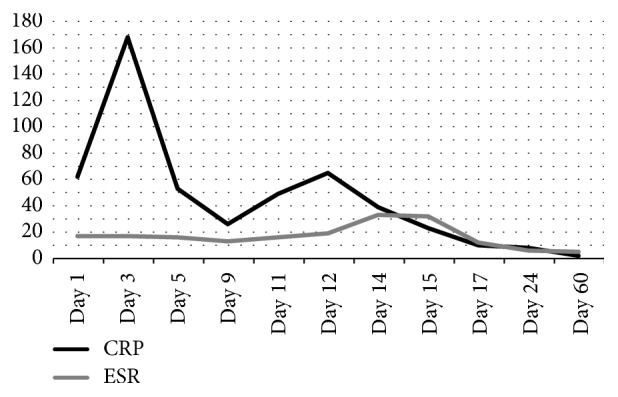
C-Reactive Protein (CRP) and Erythrocyte Sedimentation Rate (ESR).

**Figure 2:**
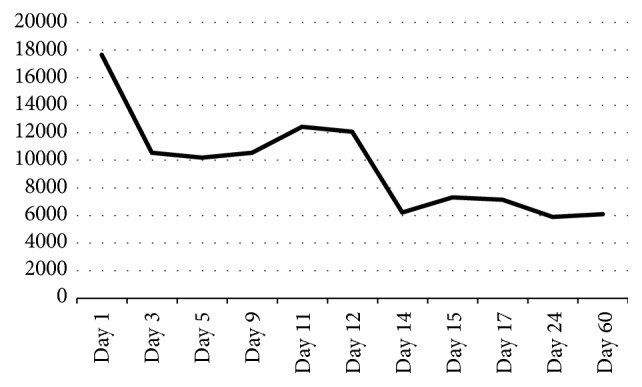
White Blood Cells (WBC) count.

Forty-eight hours after admission, laboratory analysis confirmed a type C* Neisseria meningitidis*. Since the patient was responding in a favorable way, it was decided to complete fourteen days of intravenous ceftriaxone (2 grams per day). In the first ten days, the patient achieved progress to a full range of motion (0° extension and 120° flexion), walked with two crunches, and had no signs of sepsis, no meningitis symptoms, and decreased CRP and ESV (Figures 1 and 2).

On day 10, the patient complained of knee pain. During the physical exam, extension was preserved, but he had only 90° of flexion associated with mild knee swelling. CRP and ESV on that day showed an increase compared with the previous day (Figures 1 and 2). An arthrocentesis was performed, synovial fluid was clear, and cells count was 28000. Expectant management was decided; next day the pain was minor, but ESV and CRP increased again, so it was decided to perform a new arthroscopy. Synovial fluid obtained after doing the anterolateral portal was sent for analysis; it showed 16000 cells. Arthroscopy showed normal cartilage aspect in patellofemoral, medial, and lateral tibiofemoral compartments and so were both meniscus and the cruciate ligaments. Major finding was a suprapatellar septum that was debrided. 12 liters of saline solution and a shaver were used again.

Response after the second arthroscopy was excellent; no pain or swelling was detected. The patient recovered full range of motion and walked assisted by two crunches without pain. Hospital discharge was decided on day 15 after he completed fifteen doses of 2 grams of ceftriaxone. The patient was told to complete 21 days of oral cefuroxime. He showed up for follow-up at 48 hours after hospital discharge and reported no complaints. One week later (day 24), crunches were suspended. At five weeks, he returned to work, and at 2 months he resumed sports (jogging and soccer) without complaints. WBC, ESV, and CRP have been normal since day 24 (Figures 1 and  2).

## 3. Discussion

Primary arthritis due to* Neisseria meningitidis* is rare. Serotypes B, C, D, and W135 are well known to account for major infections. Nevertheless, more frequent serotypes are B, C, and Y. Only 42 cases of primary arthritis are reported in the literature between 1980 and 2014, all of them with excellent results [5].

Although immunodeficiency could be a risk factor for* Neisseria meningitidis* infection, most cases present in immunocompetent patients as does our case. Human Immunodeficiency Virus was negative and no other signs of immunodeficiency were detected. Also, reports that include epidemiology data of septic arthritis in immunodeficiency patients do not mention* N. meningitidis* [6, 7].

In a revision performed by Straticiuc et al. in 2016, they reported 8 cases of primary arthritis due to type C* Neisseria meningitidis*, all of them in women, with the knee being affected in 6 of the 8 cases. Therefore, this is the first case reported in a male. As with other primary arthritis caused by* Neisseria meningitidis*, type C seems to have low aggressiveness for articular cartilage [8].

Standard septic arthritis treatment nowadays is arthroscopy lavage and antibiotics. The elected antibiotics depend on local epidemiology, cultures, and patient characteristics. The use of drains is controversial; we did not use one in our treatment [9]. Little evidence is published about corticoids used in the adult population for septic arthritis treatment. A recent meta-analysis performed by Farrow shows consistent positive effect when dexamethasone is added to antibiotics treatment. Nevertheless, the data is insufficient to make a recommendation. In our case, we did not use corticoids, but it was a treatment option that arose when CPR was raised in the second week of treatment, but it was discarded because there was no record in the literature for use in meningococcus knee infection [1].

Specific guidelines for the treatment of PMA do not exist; we used two arthroscopies and five weeks of antibiotic treatment. For the first two weeks, ceftriaxone was used at a dose of 2 grams per day; then cefuroxime was used for three weeks. Also, good results are reported with the use of ciprofloxacin or penicillin [4, 5, 10].

It is well known that about 40% of septic arthritis cases will need a second procedure [9], but this patient is the only case reported of primary* Neisseria meningitidis* which needed a second arthroscopy procedure. We believe that this was due to the formation of an articular septum which explains the rise of inflammatory parameters. This septum was removed in the second arthroscopy; after that the results were favorable. Another fact that supports this hypothesis is the fact that, the day before the second arthroscopy, the synovial White Blood Cells count was 28.000 and the sample of synovial fluid taken during the anterolateral approach for the second arthroscopy was 16000 White Blood Cells, much lower than the 80000 found at the time of hospital admission. This is a 43% decrease in 24 hours of evolution without any action other than antibiotics. This reinforces the theory of the intra-articular septum. We agree with other publications that* Neisseria meningitidis* has low aggressiveness for articular cartilage [8, 11].

No ESR or CRP threshold exists for septic arthritis diagnosis; both serum markers rise not only for infection but also for other inflammatory processes like gout or rheumatoid arthritis. ESR > 30 mm/hour has sensitivity of 96%, while CRP > 20 has sensitivity and specificity of 75% and 11%, respectively. In their study, Soderquist et al. report a mean CRP of 182 mg/L for septic arthritis versus a mean CRP of 101 mg/L for crystalloid arthropathy. Procalcitonin is another marker than can help with diagnosis, but levels go up as the infection is systemic rather than local. In this case, CRP was 62 mg/L and ESR was 17 mm/hr, values which are over normal limits but not as high as one might expect in septic arthritis. We consider that although it is helpful for the diagnosis, its greater value is for the follow-up during treatment [12].

No long-term results are reported in literature for this specific disease. Our patient had a fast recovery and two months after his hospital admission he resumed his daily activities. He has been asked to attend annual checkups.

## 4. Conclusion

Primary arthritis of the knee caused by* Neisseria meningitidis* is very rare. It has a very good response to antibiotics and arthroscopy procedure. Short-term follow-up and functional results are often good or excellent.
